# Progress in Medicine

**Published:** 1900-09-01

**Authors:** 


### Progress in Medicine.

### NEUROLOGY.

(*Continued from page* 353.)

**Congenital Word-blindness.**—Hinshelwood[16] records two cases, and refers to two more, of congenital letter-blindness—a difficulty in learning to read owing to some congenital defect in the visual memory centre for words and letters. In learning to read there are two distinct stages. The first stage is to store up in the visual memory the individual letters of the alphabet. When this is acquired, the individual is able to read words by spelling out aloud each letter, and thus appealing to his auditory memory; or sometimes he may simply move his lips, and thus appeal to his memory of speech movements. The second stage of learning to read consists in the gradual acquirement and storage of visual memories of words; when this is accomplished each word is recognised as a separate picture. In the first case a boy, after four and a half years at school, had not acquired the visual memory of all the letters of the alphabet. Yet his auditory memory was good; although he had not been able to learn to recognise by sight the letters and words of his little primer, he could repeat the contents of the book by heart. He could repeat the alphabet correctly, but could recognise only some of the letters by sight. He could spell rapidly and correctly most words of one syllable, such as "cat," "dog," "man," but could not recognise by sight one of these words. This failure of visual memory was for words and letters only; it did not extend to objects, persons, or places. In the second case the patient had acquired the visual memory of all the letters of the alphabet, but was very slow in acquiring that for words. After being three years at school he could recognise by sight only the most common and familiar words, so that in reading he had to pause at most of the words and spell them out, and so get the word by appealing to his auditory memory.

It is a matter of great importance to recognise these cases, for such children may be either neglected or flogged for a defect for which they are in no wise responsible. The proper method of treating such children is by patient and persistent training.

**Kernig's Sign in Meningeal Hæmorrhage.**—Kernig's sign was thought by its discoverer to be present only in affections of the pia mater, and always in inflammations of it. When the patient is recumbent the legs can be easily extended on the thighs, but when he sits up extension is rendered impossible by contraction of the flexor muscles. Recent investigations show that the sign is present in from 80 to 90 per cent. of cases of meningitis. But it is also present in meningeal hæmorrhage apart from inflammation, as shown by the following case recorded by Widel and Merklen.[17] A man, aged 32, suddenly fell from his chair unconscious. He regained consciousness in some hours without having shown any change of temperature, paralysis, or contracture. He complained only of intense headache and pain in the back. Kernig's sign was present. On the fifth day the temperature rose to 100 deg. F., and persisted at about that height until the ninth day, when death from syncope took place. At the necropsy a clot was found beneath the arachnoid, extending from the foramen magnum to the optic nerves. The spinal canal was full of liquid blood. The mechanism of the meningeal hæmorrhage escaped notice. Probably syphilis played a part. In this case, but for Kernig's sign, a meningeal lesion would not have been suspected. No doubt irritation of the meninges by the clot was the cause of the phenomenon.

**Cerebro-spinal Meningitis.**—There has been recently a small outbreak of cerebro-spinal meningitis in Dublin. Drury[18] has recorded eight cases, of whom four died and four recovered. In only one case was there a rash, a feature so typical of the great Dublin epidemic of 66-67, and this rash was not petechial, but pustular, with hæmorrhage into the pustules, and it came late in the disease. In all the cases the disease commenced suddenly, with rigidity of the neck or actual retraction of the neck, and in some cases opisthotonus. All the patients had deafness in some degree, pain in the head and limbs, and Kernig's sign. Moore said he had found that the most successful measures were leeching behind the ears and repeated blistering behind the ears. The agonising headache was relieved by a combination of acetanelid, caffein, and sodium bicarbonate. O'Sullivan said that the fluid removed by lumbar puncture was turbid but colourless, and in it the diplococcus intracellularis was found usually in pure culture. Five cases of cerebro-spinal meningitis are also recorded at Nottingham by Handford,[19] who says that they formed a striking group, with many points of resemblance. The most prominent symptoms common to all were—(1) Rapid, if not actually sudden, onset without injury or previous failure of health; (2) fever, the temperature rising at once to 102 deg.—104 deg., and very slowly subsided, sometime showing distinct and regular intermissions; (3) the intensity of the pain in the head and neck, aggravated by the slightest movement; (4) marked retraction of the head; (5) muscular hypertonicity, twitching, or rigidity; (6) extreme and rapid emaciation; (7) tendency to relapse and intermissions. The cases differ in their clinical course markedly from tuberculous meningitis. The onset is more acute, the retraction of the head much more marked, the duration of the disease much longer, and the percentage of recoveries far greater. As regards treatment morphine was given in full doses to relieve the intense pain. In three cases potassium iodide appeared to do good; in one it was evidently prejudicial, and in one without distinct effect. Morphine appeared to do good beyond the mere relief of pain.

**Facial Paralysis.**—Short[20] divides the treatment of peripheral facial paralysis of the ordinary type into local, general, and electrical. Counter-irritation behind the ear is undoubtedly of great service when the condition is seen early. During the first fortnight potassium iodide in five-grain doses should be given three times a day, and after that strychnine should be administered and continued until the muscular contraction is almost as good as on the sound side. If prolonged beyond this point there is some danger of producing over-action on the paralysed side. By far the most valuable treatment, however, is by stimulating the muscular fibres with galvanism, and so keeping up nutrition until voluntary power returns. As a rule, the battery is not applied sufficiently early; the third day is not any too early to begin. The very weakest current that will cause a contraction of the muscles should be applied. As regards prognosis, it has been thought that implication of the taste fibres points to a severe lesion, but this is certainly not always the case. Again, a prognosis based on the presence or absence of degeneration is not always to be depended on.

In the chronic form of case, especially when a little voluntary power has returned, considerable benefit sometimes accrues from the use of a small silver hook placed in the paralysed corner of the mouth, and attached by means of an elastic loop to the ear of the same side. As far as the patient is concerned, it is the paralysis of the orbicularis of the mouth which constitutes the most unpleasant feature, as it impedes speech, interferes with eating, and allows saliva to dribble away. The pull of the elastic is in direct antagonism to the action of the orbicularis, so that by its use the muscular work performed by that muscle is increased. The elastic should be just tight enough to widen the aperture of the mouth about a quarter of an inch, and it should never be worn for more than an hour at a time.

[16] Hinshelwood, Lancet, May 26, 1900. [17] Widal and Merklen, Lancet, Jan. 6, 1900. [18] Drury, Lancet, June 23, 1900. [19] Handford, Brit. Med. Jour., July 7, 1900. [20] Short, Birm. Med. Jour., Ap., 1900.

